# The effectiveness of Video-feedback Intervention to promote Positive Parenting for Foster Care (VIPP-FC): study protocol for a randomized controlled trial

**DOI:** 10.1186/s40359-018-0246-z

**Published:** 2018-08-03

**Authors:** Nikita K. Schoemaker, Gabrine Jagersma, Marije Stoltenborgh, Athanasios Maras, Harriet J. Vermeer, Femmie Juffer, Lenneke R. A. Alink

**Affiliations:** 1 Institute for Education and Child Studies, Leiden University, Leiden, The Netherlands; 2 Yulius Academy, Yulius Mental Health, Barendrecht, The Netherlands; 3 Department of Psychology, Education and Child Studies, Erasmus University Rotterdam, Rotterdam, The Netherlands

**Keywords:** Attachment, Coercion theory, Sensitivity, Foster care, Early childhood, RCT, Intervention, Video feedback

## Abstract

**Background:**

Foster children are at higher risk of the development of behavior and emotional problems, which can contribute to the development of insecure attachment bonds with their foster parents and (subsequently) to placement breakdown. Sensitive parenting might minimize the adverse effects of the behavior and emotional problems. Video-feedback Intervention to promote Positive Parenting and Sensitive Discipline in Foster Care (VIPP-FC) is an adaptation of the evidence-based Video-feedback Intervention to promote Positive Parenting and Sensitive Discipline (VIPP-SD) and aims at increasing sensitive parenting and the use of sensitive discipline strategies of foster parents. The current study is the first to examine the effectiveness of VIPP-FC.

**Methods:**

A randomized controlled trial is used with 60 foster parent-child dyads (intervention group *n* = 30, control group *n* = 30). The primary outcomes are parental sensitivity, parental disciplining, and parental attitudes towards parenting. Data about attachment (in)security, behavioral and emotional problems, neurobiological parameters, and possible confounders is additionally collected.

**Discussion:**

Examining the effectiveness of VIPP-FC contributes to the knowledge of evidence-based prevention and intervention programs needed in foster care practice.

**Trial registration:**

NTR3899.

## Background

**Fig. 1:**
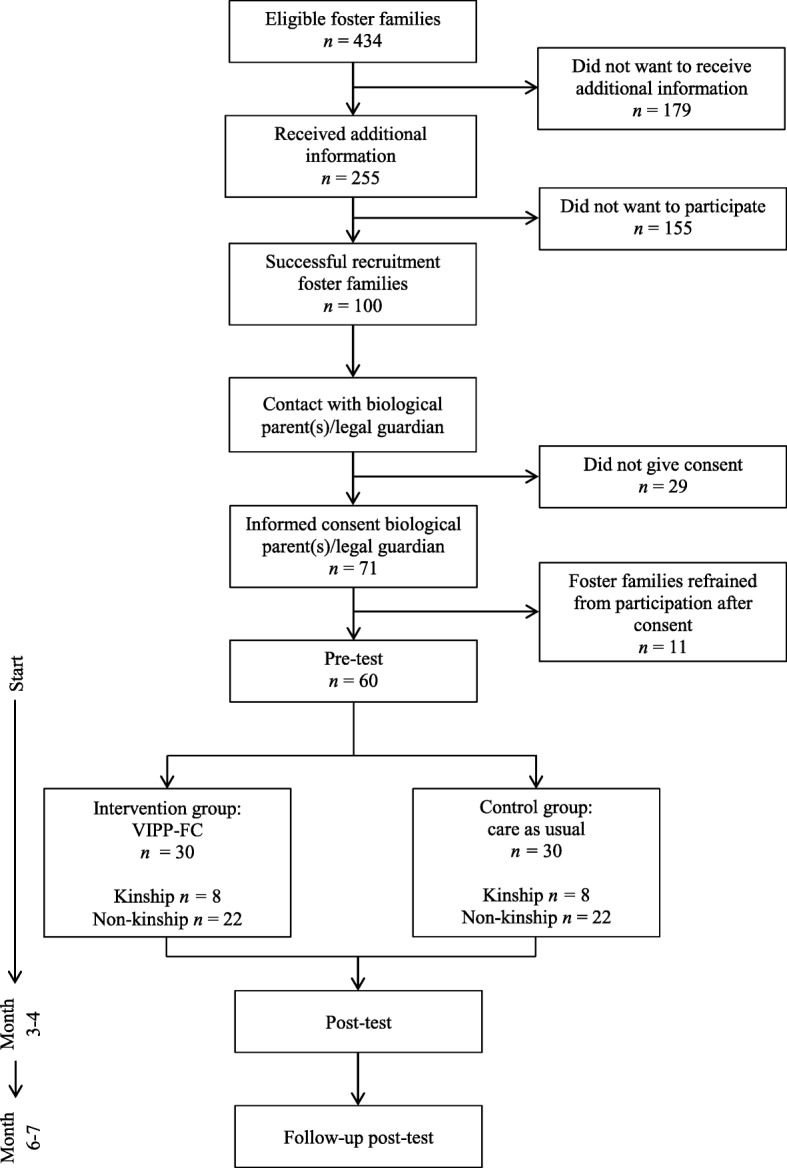
Flow diagram of study procedure

Foster children often have had adverse experiences (e.g., abuse and/or neglect) in their birth families, including separation from an attachment figure [1]. These experiences may hamper their ability to trust new adults in their lives, which subsequently can contribute to (the persistence of) behavior problems and difficulties in forming a secure attachment relationship with new parents. Meta-analytic results show that foster children are indeed twice as likely to have an insecure disorganized attachment relationship with their foster parents (36%) than children in biological families (15%) [2]. An insecure and especially a disorganized attachment relationship puts children at risk for behavior problems and psychopathology later in life [3–6]. There are concerns regarding the behavior problems of foster children which can contribute to breakdown of foster care placements [7]. Research also shows that the higher the number of placements, the higher the risk of developing psychological, behavior, and emotional problems at a later age [8].

A secure attachment relationship provides an optimal basis for children’s adaptive and resilient development [5]. A meta-analysis of intervention studies showed that increases in caregiver sensitivity were associated with increases in attachment security in the children [9]. It is therefore important that foster parents show sensitive parenting towards their foster children, provide their foster children with positive experiences, and create a nurturing environment in which the children feel secure.

It is known that parenting support that uses video feedback can help parents to recognize the behavioral signals of their child and enables them to adequately react to their child’s behavior. Video-feedback Intervention to promote Positive Parenting and Sensitive Discipline (VIPP-SD) [10–12] is an evidence-based, attachment-oriented intervention aimed to enhance parental sensitivity and sensitive discipline, by use of providing personal video feedback on recorded parent-child interactions. In order to meet the needs of foster parents and enhance the effectiveness for foster families in improving the quality of the relationship with their foster child, VIPP-SD has been adapted to VIPP Foster Care (VIPP-FC) in two ways: first, by enhancing sensitive physical contact to improve the stress regulation of both foster parents and children, and second, to support foster parents in recognizing (the absence or reduction of) behavioral signals that are specific for foster children (e.g., not crying after being physically hurt) and helping them to adequately respond to these (sometimes subtle) signals. This paper describes the adaptations of VIPP-SD to foster care and outlines the study protocol used to examine the effectiveness of VIPP-FC.

### Stress regulation

Affinitive bonds (defined as selective and enduring attachments) are formed on the basis of bio-behavioral synchrony, such as multiple hormonal, neural, autonomic, behavioral, and mental processes that coordinate to establish the parent–infant bond [13, 14]. Stress regulation plays an important role in sensitive parenting, both from the perspective of the child and the parent. Low parental nurturance can result in chronic stress for young children [15]. Early life stress, such as inadequate care and separations, is associated with long-term changes in regulation of the hypothalamic–pituitary–adrenocortical (HPA) axis. Infants who have experienced disruptions in care and who have not yet formed an attachment bond with their (surrogate) caregivers cannot benefit from the buffering effect of sensitive parenting to stress [16]. Children in foster care following involvement of Child Protective Services (CPS) within the first 2 years of life (mostly because of neglect), for example, had higher incidences of atypical patterns of cortisol production (the end product of the HPA-axis) than children without a history of CPS involvement [17–19]. Specifically, cortisol production of 55 foster children who were 20 to 60-months old decreased less across the day than the cortisol daytime levels of 104 children who had lived continuously with their biological parents [19].

There is increasing evidence that sensitive and responsive care is helpful for children with early life stress (e.g., [20]). Enhancing foster parents’ sensitivity might help normalize basal HPA axis activity of children [21]. Indeed, the effects of early life stress on the HPA axis can be reversed with interventions that support the foster parent-child relationship [22]. Children whose foster parents had received a parenting intervention (Attachment and Biobehavioral catch-up (ABC [23]) or Early Intervention Foster Care Program (EIFC [24])) showed increases in morning cortisol levels (resulting in a more normalized diurnal pattern), fewer behavior problems, increased attachment security, and fewer placement disruptions compared to a group of foster children who received care as usual.

Not only do foster children often enter their new foster home with dysregulated stress systems, foster parents are also at risk of experiencing increased stress levels. Interacting with foster children with disturbed and problematic behaviors due to their difficult life-history can be stressful for foster parents. Their increased stress levels can influence the parents’ level of sensitivity to the child. Indeed, research has shown that increased levels of maternal cortisol were related to lower parental sensitivity during parent-child interactions [20]. On the other hand, mothers who were highly sensitive during interactions with their child, had a lower heart rate indicating lower stress levels when they listened to cry sounds of babies in comparison with less sensitive mothers [25].

The forming of an affinitive bond (in different mammals such as rats, sheep, primates, and also humans) is, in addition to cortisol, related to oxytocin, a neuropeptide produced in the hypothalamus and also known as the ‘cuddle-hormone’ [26–28]. Research shows that oxytocin is related to parental sensitivity [14] and also enhances physiological and behavioral readiness for social engagement in parent-infant interactions [29]. It was found that fathers who received nasally administrated oxytocin were less hostile and offered more structured play to their child than fathers who received a placebo [30]. There are also indications that oxytocin has a decreasing effect on the amount of stress someone experiences [31]. An fMRI-study showed that the amygdala (the brain’s fear center) was less active in women who received oxytocin than in women who had not received oxytocin when hearing infant cry sounds [32]. These results indicate that oxytocin decreases the stress response of parents to children’s crying and thus may increase their responsiveness to children’s crying.

#### Positive physical contact

There is evidence that physical touch by the caregiver serves as a buffer against stress [33] and helps regulating stress in both children and adults through increased oxytocin levels and decreased cortisol levels [34]. This suggests that foster children and their foster parents can be supported in regulating stress by positive physical touch while forming an attachment bond together. From birth onwards physical touch calms down infants and children when they are in pain or discomfort [35, 36]. Foster children, however, often have had minimal experiences with positive physical touch and sometimes even experiences with negative physical touch which can result in developmental delays [34]. Fortunately, there are indications that these delays can be overcome with exposure to physical touch. Children of depressed mothers who also experienced touch deprivation benefitted from massages given by their mothers and maternal sensitivity and responsivity increased [37, 38]. It has additionally been demonstrated that play with physical contact positively correlates with oxytocin levels in parents. Mothers who often touched their baby lovingly had higher oxytocin levels afterwards [39, 40]. The same was true for fathers who interacted more playfully with their baby, for example by touching the baby with a soft toy, or by showing the baby objects. Research shows that oxytocin levels not only increase after interaction with biological children, but also with unrelated children. In fact, Bick and Dozier [41] showed that maternal oxytocin levels increased even more after playing a computer game that focused on physical contact with unrelated children than with biological children. Therefore, interventions that focus on increasing positive physical contact might help regulate stress for both the foster child as well as the foster parent.

### Behavior of foster children

Sensitive caregivers can help children to develop self-regulatory abilities [9]. These abilities can be internalized through repeated experiences of being reassured by a caregiver when children are upset and/or cry. Unfortunately, most children in foster care do not have these experiences. The absence of a familiar, trusted, and predictable caregiver leaves the child without help in regulating distress. For example, many foster children will not always show that they are in pain when physically hurt because they are often not used to being comforted and therefore the help-seeking behavior extinguishes.

The lack of self-regulatory abilities of children in foster care makes that they are often treated differently from typically developing children who grow up with their birth parents. In addition, children in foster care often have a history of maltreatment and additionally have experienced the trauma of being separated from their parents, which makes them vulnerable and susceptible to develop posttraumatic stress disorders (PTSD) and behavioral problems [7, 42–44]. To overcome the disabilities in self-regulation, it is important for foster parents to not only respond adequately to the obvious behavioral signals of the child, but to also take into account the actual situation. They should not only pay attention to behavior that they can see in the child, but also to behavior that is not there, but should be there such as showing pain or distress [44]. By providing comfort in such situations, foster parents show that the child can trust them if something is wrong. This enables foster children to adjust their expectation pattern (i.e., the internal working model of the child) to the new environment and to feel secure with the foster parents [9].

### Video-feedback Intervention to promote Positive Parenting and Sensitive Discipline in Foster Care (VIPP-FC)

VIPP-SD has been developed to enhance parental sensitivity and sensitive discipline in order to eventually promote children’s attachment security and prevent or reduce child problem behavior [11]. VIPP-SD can be used in families with children of 0 to 6 years old and consists of six intervention home-visits. The intervention method supports parents to respond sensitively to their children’s behavioral signals and to set rules and boundaries in a sensitive manner. Because of the importance of stress regulation in both children and parents and the atypical behaviors of foster children (e.g., lacking signals such as showing pain when hurt), the existing VIPP-SD program has been adapted to use in foster care (VIPP-FC) in two ways. First, a component was added that specifically focuses on increasing sensitive physical contact in order to increase oxytocin production and stress regulation in both foster children and parents. Second, a component was added that focuses on supporting foster parents in recognizing (subtle or missing) behavioral signals that are specific for foster children (e.g., not crying after being physically hurt) and how to adequately react to these signals.

### Aims and hypotheses

The current study examines the effectiveness of VIPP-FC by use of a Randomized Controlled Trial (RCT) with two groups: an intervention group receiving VIPP-FC and a control group receiving a dummy intervention. The primary goal of this study is to test the following hypothesis: VIPP-FC has a positive effect on foster parents’ sensitive parenting, sensitive discipline, and attitudes towards parenting. Additionally, this study aims to test the following secondary hypotheses: 1) VIPP-FC results in increased oxytocin production during parent-child interactions in foster parents and their foster children; 2) VIPP-FC results in better physiological stress regulation in foster parents and foster children; 3) VIPP-FC results in a reduction of behavior problems in foster children; 4) VIPP-FC results in less disorganized and more secure attachment relationships between foster children and foster parents; 5) The increase in parental sensitivity/sensitive disciplining and the decrease in child problem behavior is mediated by an increase in oxytocin production and stress regulation in foster parents and foster children, respectively.

## Methods

### Study design

We use a randomized controlled trial (RCT) with two groups: An intervention group receiving the VIPP-FC (six intervention home visits) and a control group receiving a dummy intervention (six telephone interviews). Participants are foster families living in The Netherlands. The study consists of three assessments and each assessment consists of a home visit and a visit to the laboratory. After the pretest (T1), the foster families were randomly assigned to either the intervention group or the control group. All pretests and randomization are completed. The first post-test (T2) takes place immediately after the intervention and a follow-up post-test (T3) is carried out 3 months later. Data collection for these two posttests is currently ongoing.

### Procedure

Foster families were recruited with (*n* = 56) or without mediation (*n* = 4) by nine Dutch foster care organizations spread throughout the Netherlands. In order to recruit foster families outside the range of the participating foster care organizations, advertisements of the study were published on Facebook and in a Dutch foster care magazine, and were distributed among several foster care network groups. Foster families with a foster child of 1 to 6 years of age were eligible for participation. The placement could be either kinship or non-kinship foster care, and should have been expected to last at least 6 months. Part-time or short-term crisis placements were excluded from the study. Children with severe physical disabilities, diagnosed intellectual disability (IQ < 70) and/or diagnosed autism spectrum disorder were also excluded. Lastly, twins who were placed in the same foster family could not participate in the study. If more than one child was eligible for participation within the same foster family, the most recently placed child was included, or in case of concurrent placement, the oldest child within our age range would participate.

In case of recruitment through foster care organizations, eligible foster families received a recruitment letter and a subsequent telephone call. During this call, foster parents could indicate whether they would like to receive more information about the study by (e)mail or whether they would like to make a non-committal appointment with a research assistant to receive and discuss an information brochure and an information letter in person. Foster parents who showed interest in participation without mediation of a foster care organization were also offered to receive more information about the study by (e)mail or during a non-committal appointment. To ensure blindness to study condition (intervention versus control group), foster parents were told that this study investigates various treatments to support foster parents which consist of six home visits and/or six telephone calls. After receiving more information about the study, foster parents received another telephone call within a week to ask whether they would like to participate. Because most foster parents do not have legal custody of the child, the biological parent(s) with legal custody or the legal guardian were also contacted and they received the same information as the foster parents by (e)mail or during a non-committal appointment. If both the biological parent(s)/legal guardian and the foster parents had given their consent for participation in the study, the pre-test appointments for the home visit and laboratory visit were made with the primary foster parent of the foster child. Figure 1 displays a flow diagram of the study procedure including an outline of the study design. Inclusion was finished in January 2018 and a total number of 60 foster families were included in this study.

All travel expenses are compensated and both foster parent and the child receive a small gift after completing every assessment. As a compensation of their time and effort foster parents receive a financial reimbursement of €100 for their participation in the study.

Participating foster families in either the intervention or control group are not prevented to use medical drugs. Both also receive the care as usual provided by foster care organizations. If needed, foster families assigned to the control group can receive additional treatment (as part of the care as usual) during the study period. All additional treatments in both groups are documented. If necessary, type and amount of additional care and treatment can be controlled for in analyses.

This study was approved by the Medical Ethics Committee of the Maasstad Hospital in Rotterdam, The Netherlands. The trial is registered in the Netherlands Trial Register (NTR; Trial ID: NTR3899).

### Study sample

A total of 434 foster families were eligible for participation (Fig. 1). One hundred seventy families (41.2%) did not want to receive additional information and 155 families (35.7%) did not want to participate after receiving additional information, resulting in a successful recruitment of 100 foster families (23.0%). The biological parents with legal custody or the legal guardian of 29 (6.7%) children did not give consent for participation. Additionally, 11 foster families (2.5%) refrained from participation after giving informed consent, mostly due to personal circumstances. A final sample of 60 families (13.8%) was enrolled.

The children were on average 3.63 years old (*SD* = 1.35, range: 1 to 6) at pretest, 27 (45.0%) are boys, and 73.3% of the children are placed with a non-kinship foster family. All foster parents, of which 50 (83.3%) foster mothers, participating in the study are the primary caregiver of the child with a mean age of 45.43 years (*SD* = 7.42, range: 31 to 61). The foster parents have on average 1.74 (*SD* = 0.83, range: 1 to 4) foster children and on average 1.87 (*SD* = 1.39, range: 0 to 5) biological children.

### Randomization

The random assignment to the VIPP-FC intervention or control group was done using a computer-generated blocked randomization sequence, stratified by kinship or non-kinship foster care and with a block size of 10 foster families. Group allocation was performed after the pretest and before the start of the intervention. Participating foster families are blind to condition and all data will be coded by independent researchers who are blind to the condition of foster families.

### Sample size and power

Recent meta-analytic results of 12 studies using an RCT-design investigating the effects of VIPP-SD on increased caregiver sensitivity showed a combined effect size of *d* = 0.47 and a combined effect size of *d* = 0.26 for reduced problem behavior in the children [11, 12]. To test the effectiveness of VIPP-FC on foster parents’ sensitivity and sensitive discipline with a repeated measures design with *α* = 0.05 and a study sample of 60 foster families the statistical power is adequate (0.86; repeated measures ANOVA within-between interaction, G*Power 3.1.9.2).

### Video-feedback Intervention to promote Positive Parenting and Sensitive Discipline in Foster Care (VIPP-FC)

#### Theoretical background

VIPP-FC is an adaptation of VIPP-SD with specific components to use in foster families. VIPP-SD is based on attachment theory [45, 46] and coercion theory [47].

Attachment theory states that every child develops an attachment relationship with their primary caregiver. This caregiver provides a secure base from which the child can explore the world, and is also a safe haven where the child can return to in times of need. The quality of the attachment relationship depends on the caregiver’s availability and on how he/she responds to signals of the child. In VIPP-SD parents are supported to show more sensitive responsive behavior toward their child by observing and interpreting the child’s signals accurately and respond to these signals promptly and adequately [45].

Patterson’s coercion theory is based on the social learning theory of Bandura [48] and states that children’s externalizing behavior is reinforced and enlarged when the child reacts to the caregiver’s rules and demands with negative behavior, and thus forces the caregiver to adjust his/her rules and demands, while the caregiver concedes and lowers his/her rules and demands [47]. The child ‘learns’ that this strategy of using negative behavior works and will use it again in the future. The absence of the reinforcement of desired (positive) behavior combined with inconsistent disciplining contribute to the development of externalizing behavior (e.g., aggression and hyperactivity) of the child. The opposite of inconsistent disciplining is sensitive disciplining and induction: to offer warmth, support, and responsivity [45], and to set rules and boundaries in a sensitive manner, to prohibit negative behavior, and explain why something is not allowed (i.e. induction) [49] at the same time. Negative and inconsistent limit-setting can be considered as being not adequately attuned to the child’s behavior and thus as insensitive caregiving. Both attachment theory and coercion theory emphasize that insensitive caregiving can contribute to problem behavior in children. Increasing parental sensitivity can, on the other hand, prevent or decrease children’s problem behavior.

#### Structure and training

The intervention consists of six home visits: The first four sessions are biweekly and there is an interval of approximately 3 weeks between sessions four and five and sessions five and six. During each home visit, the participating foster parent (primary caregiver) and child are filmed during daily situations for 10 to 30 min, such as playing, mealtime or reading a book together. The foster parent is asked to behave and respond to the child as they would normally do and the intervener does not intervene during filming. After filming, the intervener gives personal video feedback on the interactions between foster parent and child of the previous home visit, with a focus on positive interactions and sensitive discipline. This video feedback is prepared by the intervener during the interval between two home visits. During the discussion, the intervener acknowledges the foster parent as an expert of the foster child and foster parent and intervener also talk about general child development, sensitive disciplining strategies, and specific behaviors often seen in foster children (i.e., indiscriminate friendliness). Apart from general information about parenting and child development, the first four sessions have different specific themes regarding sensitivity and sensitive discipline. The last two sessions are booster sessions, during which all themes are repeated.

The interveners are foster care professionals working at one of the participating foster care organizations or researchers involved in the research project. All interveners have completed an extensive training in VIPP-SD and VIPP-FC, using a manual which contains the description of each session’s structure, themes, tips, and exercises. In order to gain intervention fidelity, every intervener fills out a logbook for each home visit in which the details of the visit are described. Supervision is given to the interveners during the preparation of at least three home visits to obtain intervention fidelity.

#### VIPP-SD themes for parental sensitivity

During the first home visit, the intervener shows the difference between exploration (i.e., playing) and attachment behavior (i.e., contact seeking) of the child, and explains the different parental responses these behaviors require. The second home visit focuses on ‘speaking for the child’ which promotes the accurate observation of (subtle) child signals by articulating the child’s facial and other non-verbal expressions on video. Explaining the importance of prompt and adequate responses to child signals by means of a so-called sensitivity chain is discussed and shown during the third home visit. During the fourth home visit, the intervener shows and encourages parental affective attuning to positive and negative emotions of the child.

#### VIPP-SD themes for sensitive discipline

Inductive discipline and distraction are the sensitive discipline strategies that are discussed during the first home visit. Both can be used as responses to difficult behavior or conflict situations. Using inductive discipline, i.e., explaining why something is commanded or forbidden, aims to promote empathy in the child by explaining other people’s interest and perspective. During the second home visit, the intervener discusses the importance of the use of positive reinforcement by praising the child for positive, desirable behavior while ignoring the child’s attempts to get attention for negative, unwanted behavior. The third home visit focuses on the use of a sensitive time-out. This type of time-out can be used to prevent temper tantrums to escalate and to make the situation bearable for the foster parent. The last sensitive discipline theme is empathy for the child, combined with consistent use of disciplining strategies and clear boundaries.

#### VIPP-FC additional themes

The first additional theme targets the improvement of stress regulation. To address this theme, in each home visit an extra situation is added during which foster parent and child are asked to play a (singing) game with physical contact while being filmed by the intervener. During video feedback the intervener discusses the importance of sensitive physical contact for stress regulation and helps the foster parent to recognize and to sensitively respond to the child’s signals during these situations. To encourage foster parents to have more daily positive physical contact, they receive a booklet with different types of physical interaction games.

The second theme supports foster parents in how to respond in a sensitive manner to missing or subtle behavioral signals. During video feedback the intervener discusses how possibly disturbed behavior of foster children can be understood and why it is important to adequately respond to these behaviors. During video feedback the intervener helps foster parents to recognize missing or subtle signals and shows them how they can reinforce the child’s (subtle) signals to express attachment behaviors.

### Dummy intervention

Foster families in the control group receive a dummy intervention of six telephone calls to ensure that the number of contact moments with interveners is the same for the intervention and the control group. The research assistant performing the telephone calls follows a protocolled semi-structured interview. During the calls, foster parents are invited to talk about topics regarding the general development of their foster child (e.g., playing alone and with other children, sleeping behavior, eating behavior, etc.), but no specific information or advice about typical or atypical child development or parenting is given.

### Primary outcome measures

#### Parental sensitivity

Parental sensitivity is observed during two free play episodes, one with and one without toys. During the free play episode with toys the foster parents and children are given several toys to play with for 5 min. During the free play episodes without toys no toys are given and foster parents are instructed to play together with their child for 5 min. They can decide for themselves what to do during this episode.

Parental sensitivity is coded using slightly adapted Ainsworth scales for *sensitivity* and *non-interference* [50] (Mesman: Ainsworth's observation scale for sensitivity vs. insensitivity, unpublished) to be able to use the scales for the interaction of parents with older children (instead of infants). Sensitivity is defined as observing and interpreting the signals of the child accurately and responding to these signals promptly and adequately [45]. Sensitivity is scored on a nine point scale, ranging from ‘highly insensitive’ with rare or absent sensitive responses to ‘highly sensitive’ with the parent responding sensitively to the child’s signals almost continuously throughout the episode. Non-interference is defined as the child being able and allowed to take the lead in the interaction. Non-interference is scored on a nine point scale, ranging from ‘highly interfering’ with the parent unnecessarily interfering with the child’s behavior and intentions almost throughout the whole episode to ‘not at all interfering’ with the child leading the interaction.

#### Parental disciplining

Parental disciplining is observed during a Don’t Touch task and a Clean Up task. During the Don’t Touch task the foster parents are given a bag of attractive toys that make sounds, are colorful and/or can be used interactively. They are instructed to take the toys out of the bag, put them in front of the children, and to refrain their children from touching the toys. After 1 min, the children can play with the least attractive toy (i.e., a stuffed animal rabbit). After another minute, the children can play with all the toys. During the Clean Up task the foster parents and children are given several bags and boxes and are asked to clean up the toys they played with during the free play with toys episode (used for coding parental sensitivity) described above. The task is finished if all the toys are put away. The researcher ends the episode if the toys are not completely cleaned up yet after 5 min.

Parental disciplining is coded using three scales: *harsh physical discipline, verbal overreactive discipline* [51, 52], and the Erickson scale for *supportive presence* [53, 54]. Harsh discipline is defined as using unnecessary force to get the child to clean up or to prevent the child from touching a toy when he/she is not allowed to do so. Physical force that is used to reinforce a command or prohibition is also coded as harsh discipline. Examples are slapping, pulling the child’s arm, forcefully taking away toys from the child. The physical impact on the child of the harsh action should be noticeable, e.g., movement of body, and/or shock/discomfort is expressed (non)verbally. Harsh discipline is scored on a five point scale, ranging from no physical harsh acts to predominantly physical harsh acts during the episode, with at least one act of physical punishment. Verbal overreactive discipline is defined as verbally expressing irritation and anger towards the child. Tone of voice is coded here, not the content of the verbal statements. Examples are yelling, screaming, and an impatient, irritated, unkind and/or angry tone. Verbal overreactive discipline is scored on a five point scale, ranging from no verbal overreactivity to predominantly verbal overreactivity with the parent expressing his/her irritation and/or anger almost continuously throughout the episode. Both harsh discipline and verbal overreactive discipline are reverse coded so that a higher score indicates more sensitive discipline skills. Supportive presence is defined as verbally of nonverbally expressing positive regard and emotional support. Examples are reassuring the child when he/she finds the task difficult, and moving closer to the child to give him/her a physical sense of support. Supportive presence is scored on a seven point scale, ranging from the parent completely failing to be supportive to the child because the parent does not show interest in how the child behaves and performs the task, to the parent offering positive reinforcement and emotional support throughout the whole episode.

#### Attitudes of foster parents towards parenting

The foster parents’ *attitudes toward sensitivity and sensitive discipline* are assessed using a questionnaire regarding their attitudes towards parenting (Bakermans-Kranenburg & Van IJzendoorn: Vragenlijst voor kennis en attituden over de opvoeding. [Questionnaire concerning knowledge and attitudes toward parenting], unpublished). Foster parents are asked to rate 43 statements about their attitudes on a five point Likert scale ranging from totally disagree to totally agree (e.g., “In my opinion, I should praise my child at least once every day”).

### Secondary outcome measures

#### Quality of the attachment relationship

Attachment security and disorganization are assessed using the Strange Situation Procedure (SSP; [45]). The MacArthur Preschool Attachment Classification System (PACS) is used to categorize the foster children in one of four attachment classifications, i.e., *secure, insecure avoidant, insecure ambivalent, or insecure disorganized* (Cassidy, Marvin, the MacArthur Working Group on Attachment: Attachment organization in 2 1/2 to 4 1/2 year olds: Coding manual, unpublished).

#### Behavioral and emotional problems

The children’s *behavioral and emotional problems* are assessed using the Child Behavior Checklist (CBCL [55, 56]) and the Assessment Checklist for Preschoolers (ACP-Short Form [57–59]), both filled out by the foster parent.

#### Indiscriminate friendliness

*Indiscriminate friendliness*, being child behavior defined as being friendly and compliant towards all adults including strangers [60, 61], is assessed with the Indiscriminate Friendliness Questionnaire [62] filled out by the foster parent and with an observation using the Stranger at the Door procedure; SATD [63]. To gain more insight in the severity of indiscriminate friendliness we developed a more elaborate coding system for the SATD than Zeanah et al. [63]. In addition to coding whether or not a foster child is willing to leave with a stranger, we also code if the child hesitates and/or displays social referencing (e.g., seeking proximity) towards the foster parent when invited to leave with a stranger.

#### Neurobiological and other parameters

*Salivary alpha-amylase (sAA)* production, a proxy of autonomic nervous system (re)activity, of foster parents and children is measured during the laboratory visit (three times: before and directly after the SSP, and 30 min after the SSP had ended).

Diurnal *cortisol* levels of foster parents and children are measured in saliva collected at home (four times: immediately after waking up, 30 min after waking up, between 1 and 3 pm, and between 5 and 6 pm). A hair sample is also collected to obtain a measure of the *cortisol* production of the last months. Hair grows approximately 1 cm per month, which makes it possible to determine fluctuation in cortisol production over the past few months. During the home visit of each assessment (i.e., pre- post-, and follow-up post-test), a strand of about 100 hairs of both foster parents and children is collected from the middle of the back of the head [64] and stored in a dark filing cabinet.

*Oxytocin* production of foster parents and children is measured in saliva collected before and after a computer task that elicits physical interaction between foster parent and child [65] during the laboratory visit of the pre-, post-, and follow-up post-test.

#### Possible confounders

Possible confounders regarding foster family and child characteristics, such as type of foster care placement (kinship vs. non-kinship), duration of placement, family composition, age, sex, ethnicity, social economic status (SES), and support and interventions received since the foster care placement are measured with a questionnaire.

## Discussion

Children in foster care are a vulnerable population. They are more likely to show an insecure attachment than children in biological families [2], which can contribute to behavior problems and psychopathology later in life [3–5]. There is increasing evidence that sensitive and responsive parenting is helpful for children with early life stress such as the stress foster children have experienced (e.g. [8]).

Several randomized controlled trials have been conducted in the USA to meet the need for parental sensitivity-focused, evidence-based prevention and intervention programs for this high-risk population. Examples of effective interventions for foster care are Attachment and Biobehavioral Catch-up (ABC; [23]), Multidimensional Treatment Foster Care for Preschoolers (MTFC-P; [21]), Parent-Child Interaction Therapy (PCIT; [66, 67]), Promoting First Relationships (PFR; [68, 69]), and Parent Management Training-Oregon Model (PMTO; [70]). However, little is known about the effectiveness of these or comparable prevention and intervention programs in the Netherlands. MTFC-P, for example, did not result in the same improvements in a Dutch foster care population as in the US [71]. Video-feedback Intervention to promote Positive Parenting and Sensitive Discipline (VIPP-SD) is one of the few evidence based intervention programs in The Netherlands in other populations than foster care [11, 12, 72]. In order to meet the need for evidence-based intervention programs in the Dutch foster care system, the current study aims to provide insight into the effectiveness of an adaptation of the VIPP-SD for foster care. VIPP-FC is a short intervention, with only six intervention home-visits over a period of 3 to 4 months.

There are several vulnerabilities regarding the study design. First, because informed consent of both foster parents as well as biological parents with legal authority or the legal guardian was needed, it took some time before all forms for informed consent were signed. Subsequently, the study itself takes approximately 6 to 7 months to complete per foster family. During this time period many things can change. For example, visitation arrangements with the biological parent might change, which can cause stress in the child and the foster parents. Therefore the researchers are as flexible and as adaptive as possible by, for example, meeting the families at their houses at any day or time in order to complete the assessments. Additionally, the researchers invest in a good working alliance with the foster care professionals throughout the different organizations.

A strength of this study is the close collaboration with different foster care organizations. The VIPP-FC training for foster care professionals was offered to all participating organizations in this study. A total of 88 foster care and health care professionals throughout The Netherlands were trained in this intervention. In case the results will show that VIPP-FC is effective in increasing foster parent’s sensitivity and sensitive discipline, organizations can immediately continue the implementation of this new intervention as a component of their care to foster families.

In conclusion, foster children are vulnerable for developing behavioral and emotional problems, which can contribute to the development of insecure attachment bonds with their foster parents and placement breakdown. In this study VIPP-FC aims to increase foster parents’ sensitivity and, use of sensitive discipline strategies towards their foster child and to have a positive effect on foster parents’ attitudes towards parenting. If VIPP-FC is effective, it will be made available for broad-scale implementation in (clinical) practice in the Netherlands.

## Abbreviations

ABC: Attachment and Biobehavioral Catch-up
ACP: Assessment Checklist for Preschoolers
CBCL: Child Behavior Checklist
CPS: Child Protective Services
EIFC: Early Intervention Foster Care Program
HPA: Hypothalamic-pituitary-adrenocortical
MTFC-P: Multidimensional Treatment Foster Care for Preschoolers
NTR: Netherlands Trial Register
PACS: Preschool Attachment Classification System
PCIT: Parent-Child Interaction Therapy
PFR: Promoting First Relationships
PMTO: Parent Management Training-Oregon Model
PTSD: Posttraumatic stress disorder
RCT: Randomized Controlled Trial
sAA: Salivary alpha-amylase
SATD: Stranger at the door
SES: Social economic status
SSP: Strange Situation Procedure
VIPP-FC: Video-feedback Intervention to promote Positive Parenting and Sensitive Discipline in Foster Care
VIPP-SD: Video-feedback Intervention to promote Positive Parenting and Sensitive Discipline
